# Perturbation in kidney lipid metabolic profiles in diabetic rats with reference to alcoholic oxidative stress

**DOI:** 10.4103/0971-4065.57106

**Published:** 2009-07

**Authors:** K. R. Shanmugam, C. H. Ramakrishna, K. Mallikarjuna, K. Sathyavelu Reddy

**Affiliations:** Division of Molecular and Exercise Physiology, Department of Zoology, Sri Venkateswara University, Tirupati-517 502, A.P, India; 1 Exercise Biochemistry Lab, Taipei Physical Education College, Taipei City, Taiwan

**Keywords:** Alcohol, diabetes, lipid metabolic profiles, rats

## Abstract

Diabetes is a major threat to global public health, and the number of diabetic patients is rapidly increasing worldwide. Evidence suggests that oxidative stress is involved in the pathophysiology of diabetic complications and alcoholic diseases. The aim of this study is to find out the impact of alcohol on lipid metabolic profiles in kidney tissue under streptozotocin induced diabetic condition. No study has been reported so far on the effect of alcohol on diabetic condition and also with reference to lipid metabolic profiles. Hence, the present study has been designed to elucidate the impact of alcoholism on diabetic condition. Male wistar strain albino rats were randomly divided into four groups: control (saline treated) NC, alcohol-treated (At), diabetic control (DC), and alcohol-treated diabetic rats (D+At). In alcohol-treated diabetic rats, we observed high levels of MDA, total cholesterol, triglycerides, phospholipids and also high levels of blood glucose than other groups. Moreover, degenerative changes of renal cells in alcohol-treated diabetic group were maximized by administration of alcohol as evinced by histopathological examination. This study suggests that alcohol consumption could be an aggravation factor which contributes for the formation of free radicals in diabetic condition. Therefore, consumption of alcohol during diabetic condition is harmful.

## Introduction

Diabetes mellitus is characterized by hyperglycemia together with biochemical alterations of glucose and lipid peroxidation.[1] Lipid peroxidation, a free radical-related process, is an uncontrolled, self-enhancing process causing disruption of membranes, lipids and other cell components. Unlimited lipid peroxidation (LP) could be one of the main factors in the pathogenesis of diabetic complications. This pathology is often related to the release of free radicals and cause oxidative stress.[2] During reoxygenation, hypoxantine-xantine oxidase and arachidonic acid pathways are important sources of free oxygen radicals, which damage lipid membranes, and lead to cytolysis and cell death.[3] Accumulation of lipids in diabetes is mediated through a variety of derangements in metabolic and regulatory processes, especially insulin deficiency, thereby rendering the diabetic patient more prone to hypercholesterolemia and hypertriglyceridemia.[4] One of the major pathogenesis of lipid metabolism disturbances in diabetes is the increased mobilization of fatty acids from adipose tissue and secondary elevation of free fatty acid level in the blood.[5] Lipid abnormalities such as hypercholesterolemia, hypertriglyceridemia, hyperphospholipidemia[6] and fatty acid distribution changes are common in diabetic patients.[7]

Alcohol is one of the oldest and most widely used psychoactive drugs on earth. Its use pre-dates recorded history and may go back as far as the Paleolithic age, around 8000 BC.[8] It has been proposed that free radical generation processes and lipid peroxidation products may play a focal role in the mechanism by which alcohol exerts its toxic effects in the liver and other tissues.[9] Renal tubular dysfunction, including impairment in renal acidification is reported in some of alcoholics.[10] There are limited reports on the effect of alcohol during diabetic condition. Numerous studies have demonstrated a J-shaped relationship between alcohol consumption and diabetes.[11,12] Moderate consumption of alcohol is medically beneficial and socially acceptable,[13] as moderate alcohol consumption also beneficially affects insulin sensitivity and glucose metabolism.[14] However, excessive consumption of alcohol leads to complex health problems, because alcohol may alter glycemic regulation and potentially aggravate the cardiovascular, neurological, and immunosuppressive changes in patients with diabetes mellitus. The previous lack of data regarding the influence of alcohol in diabetes resulted in the doctrine that alcohol consumption was universally detrimental in diabetic patients.[15] Few reports on alcohol treatment to diabetic rats with reference to total cholesterol, triglycerides, phospholipids and lipid peroxidation levels were reported With reference to kidney tissue, there were no reports. Hence, the present study has been carried out to know the effect of alcohol on all these parameters during diabetic condition.

## Materials and Methods

### Animals

Wistar strain albino rats of male sex weighing 180 ± 20 g were obtained from Indian Institute of Sciences Bangalore. The rats were housed in clean polypropylene cages having six rats per cage and maintained under temperature controlled room (27 ± 20°C) with a photoperiod of 12-h light and 12-h dark cycle. The rats were fed with a standard rat pellet diet and water *ad libitum*.

### Chemicals

All the chemicals used in the present study were of Analar Grade (AR) and obtained from the following scientific companies: Sigma (St. Louis, MO, USA), Fischer (Pitrsburg, PA, USA), Merck (Mumbai, India), Ranbaxy (New Delhi, India), and Qualigens (Mumbai, India).

### Induction of diabetes

Streptozotocin (STZ) in citrate buffer (pH 4.5) was administered intraperitoneally (i.p.) at a single dose of 50 mg/kg to groups III and IV.[16] After injection, they had free access to food and water and were given 15% glucose solution to drink overnight to counter hypoglycemic shock. Diabetes in rats was identified by moderate polydispia and marked polyuria. From the third day onwards, fasting blood samples were collected from the rats by tail vein and the blood glucose was measured by Accu Chek Sensor comfort glucometer (Manufacturer-Roche Germany) to know the induction of diabetes. Rats with blood glucose level of 250 mg/dl or higher were considered to be diabetic. Rats were allowed to acclimatize the diabetic condition for one week. After one week, the rats with hyperglycemia (blood glucose level 250 mg/dL) were selected and used for the study. On eight day the treatment was started and continued for 30 days.

### Grouping of animals

The rats were divided into four groups of six rats in each group and treated as follows:

1. Normal control (NC): This group of rats received saline (0.9%), for a period of 30 days.
2. Alcohol treatment (At): This group of rats received absolute alcohol orally with a dose of 2 g/kg body weight via orogastric tube for 30 days.
3. Diabetic (STZ 50 mg/kg body weight) (DC): Streptozotocin was given intraperitonially for the induction of diabetes for this group.
4. Diabetic plus alcohol treatment (D+At): Diabetic rats received alcohol.

### Body weight changes

The body weight changes were calculated by recording the body weights of all experimental groups at interval for 30 days.

### Tissue processing

The animals were sacrificed after 24 h of the last treatment by cervical dislocation. The kidney was excised at 4°C, washed with ice cold saline and blotted. After the atria and blood vessels were trimmed, the kidney were immediately immersed in liquid nitrogen and stored at -80°C for further biochemical analysis. The selected lipid metabolic profiles such as lipid peroxidation (MDA), total cholesterol (TC), triglycerides (TG) and phospholipids (PL) levels were monitored by the methods of Ohkawa *et al.,* (1979), Liebermann Bernhard reaction as described by Natelson (1971), Zilversmidth and Davis (1950) respectively. The experiments were carried out in accordance with the guidelines and protocol approved by the Institutional Animal Ethics Committee.

### Statistical analysis

The data has been analyzed by using SPSS (Version 13.5; SPSS Inc., Chicago, IL, USA) and M.S. Office, Excel Software for the significance of the main effects (factors), and treatments along with their interactions. The data has been compared using one way ANOVA with Dunnett's multiple comparison test and differences were considered significant at *P* < 0.001.

## Results

In alcohol-treated rats, blood glucose levels were increased. With alcohol treatment in diabetic rats, blood glucose levels were drastically increased than that of normal control, alcohol-treated and diabetic rats [Table 1].

**Table 1:** Blood glucose levels (mg/dl), body weights (grams) and kidney weights (grams) of normal control, alcohol treatment (2 gm/kg body weight), diabetic (50 mg/kg body weight) and diabetic + alcohol treatment rats

	Blood glucose (mg/dl)		Body weight (grams)		Kidney weight (grams)
		
	0 day	30 day	0 day	30 day	
Normal control	81 ± 1.41	94 ± 2.8 (+16.049)	195 ± 9.66	215 ± 14.28 (+10.256)	1.55 ± 0.06
Alcohol treated	81 ± 1.414 (+0.00)	121 ± 5.811* (+49.592)	205 ± 5.2 (+5.128)	225 ± 6.05* (+15.384)	1.47 ± 0.042 (-5.161)
Diabetic control	253 ± 3.53 (+212.345)	269 ± 15.6* (+232.098)	200 ± 7.36 (+2.564)	150 ± 6.83* (−23.076)	1.70 ± 0.04 (+9.677)
Diabetic+alcohol treatment	256 ± 4.09 (+216.049)	377 ± 14.945* (+365.432)	205 ± 4.47 (+5.128)	125 ± 3.764* (−35.897)	1.71 ± 0.04 (+10.322)

All the values are mean ± SD of six individual observations, Values in the parentheses denote per cent change over normal control,

* Significant at *P* < 0.001 with Normal control.

A significant elevation in TC, TG, PL and MDA levels were observed in the alcohol treated and diabetic control rats. However, with alcohol treatment in diabetic rats, we observed very high levels of TC, TG, PL and MDA. [Tables 2 and 3].

**Table 2:** Changes in MDA content and total cholesterol levels in the kidney tissue of normal control, alcohol treated, diabetic control, diabetic + alcohol treated rats

Parameter	Normal control	Alcohol treated	Diabetic control (STZ)	Diabetic + alcohol treatment
MDA^a^	45.6667 ± 3.9168	61.33* ± 4.700 (+34.307)	72.3750* ± 3.6116 (+58.487)	80.792* ± 5.256 (+76.919)
Total cholesterol^b^	77.6720 ± 2.8997	101.005* ± 2.351 (+30.040)	104.8133* ± 3.5583 (+34.943)	111.477* ± 3.034 (+43.522)

All the values are mean ± SD of six individual observations,

a Values are expressed in μ moles of malondialdehyde formed per gram wet weight of the tissue

b Values are expressed in mg of total cholesterol per gram wet weight of the tissue. Values in the parentheses denote percent change over normal control

* Significant at *P* < 0.001 with Normal control.

**Table 3:** Changes in triglyceride content and phospholipid content in the kidney tissue of normal control, alcohol treated, diabetic control, diabetic + alcohol treated rats

Parameter	Normal control	Alcohol treated	Diabetic (STZ) (DC)	Diabetic + alcohol treatment
Triglyceride^a^	1.7302 ± 0.1091	3.954* ± 0.539 (+128.554)	5.0485* ± 0.1116 (+191.79)	6.278* ± 0.388 (+262.890)
Phospholipid^b^	18.3328 ± 4.0326	34.141* ± 4.261 (+86.216)	46.6462* ± 2.4274 (+15.445)	54.999* ± 6.587 (+199.983)

All the values are mean ± SD of six individual observations

a values are expressed in mg of triglycerides/gram wet weight of the tissue

b Values are expressed in mg of Phospholipids/gram wet weight of the tissue, values in the parentheses denote per cent change over normal control

* significant at *P* < 0.001 with normal control. The study was performed according to all the guide lines and protocols approved by the Institutional Animal Ethics Committee (Regd. No. 438/01/a/CPCSEA/ dt.17.07.2001) in its resolution number 9/IAEC/SVU/2001/dt. 4.03.2002).

**Figure 1:**
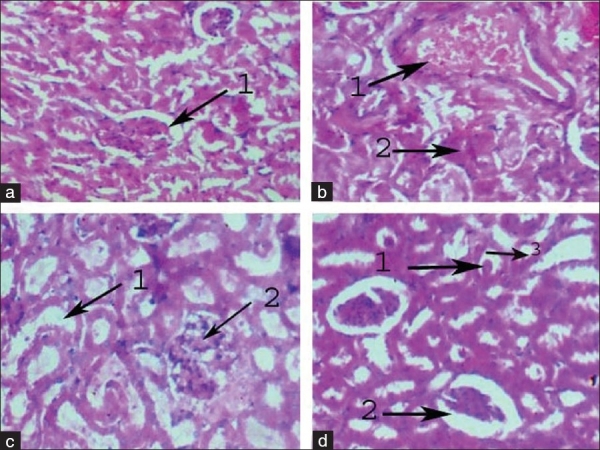
(a) Photo micro graph of normal control kidney renal parenchyma showing normal architecture with normal Bomens capsule. (b) Photo micro graph of alcohol-treated (At) kidney showing 1. Severe necrosis of the cells 2. Severe congestion of blood vessels. (c) Photo micro graph of diabetic control kidney showing 1. Severe tubular degeneration, 2. Focal necrosis of tubules. (d) Photo micro graph of diabetic + alcohol treated (D+At) rat kidney showing 1. Hyaline casts, 2. Dilatation of Bowmens capsule, 3. Tubular damage.

In STZ-induced diabetic control rats, severe tubular degeneration, degeneration of glomeruli, focal necrosis of tubules, cystic dilatation of tubules and fatty infiltration in the kidney tissue. The above pathological changes were enhanced in alcohol-treated diabetic rats and also dilatation of bowmen's capsule, hyaline casts were observed. The histological picture was worse in diabetic rats with alcohol treatment. Its shows that alcohol aggravates the production of free radicals in diabetic rats, so the kidney tissue was damaged in this group [Figure 1].

## Discussion

The present study was designed to investigate the effect of alcohol on blood glucose levels and on antioxidant enzymes in STZ induced diabetic rats. Results of this study demonstrated that alcohol induced significant increase in the blood glucose levels in the STZ-induced diabetic rats. In the current investigation, blood glucose levels were elevated in STZ-induced diabetic rats due to an oxidative stress produced in the pancreas, and a single strand break in pancreatic islets DNA.[17] Where as in alcohol treatment alone (At) and in combination treatments (D+At) blood glucose levels were increased. A number of investigations have reported that alcohol treatment in diabetic rats elevates the blood glucose levels. Heavy alcohol consumption by the diabetic patients showed elevated levels of glucose due to oxidative stress and auto-oxidation of glucose.[18] Increased insulin secretary responses and enhanced glucose disposal rates have been observed after moderate alcohol ingestion in subjects with type 2 diabetes.[19]

In the current study, we found raised total cholesterol in diabetic group. The most common lipid abnormalities in diabetes are hypercholesterolemia and hypertriglyceridemia.[20] Increased level of total cholesterol may be due to higher cholesterol biosynthesis and/or liposysis.[21] The increased level of cholesterol in kidney is due to the decreased level of HDL-cholesterol.[22] These results confirm that there is a strong correlation between oxidative stress and diabetes. A variety of derangements in metabolic and regulatory mechanisms due to insulin deficiency are responsible for the observed accumulation of lipids.[23] Marked alterations in lipid metabolism have been reported in chronic alcohol fed rats.[24] Reports show that chronic alcohol ingestion results in moderate hypercholesterolemia and hypertriglyceridemia.[25] Chronic administration of alcohol increases tissue cholesterol and FFA. Increase in cholesterol may be due to the accumulation of fat in tissues and excess cholesterol leaking out in to the blood and entering the kidney. Alcohol treatment to diabetic rats may favor the excess lipolysis in kidney, so we observed high levels of total cholesterol. The observed hypercholesterolemia in alcohol-treated diabetic rats in the present study agrees with findings of the earlier reports.[26,27,6] Wei *et al*.[28] Nakanishi *et al*.[29] and Fukui *et al*.[30] reported that alcohol consumption during diabetes increases the total cholesterol in men.

Phospholipids are vital part of biomembrane rich in PUFA, which are susceptible substrate for free radicals, such as O2•^−^ and OH^−^ radicals. In our study, phospholipids level was increased in diabetic group. Earlier reports also confirmed increased levels of phospholipids in tissues and serum in STZ induced diabetic rats.[31] Elevated levels of phospholipids in kidney tissue have been observed in diabetic rats which may result in a number of deleterious effects due to accumulation of H_2_O_2_ and abnormal fatty acid metabolism.[32] Phospholipid level was significantly elevated in kidney tissue of alcohol treated rats. They are the primary targets of peroxidation and can be altered by alcohol.[33] These elevated levels can result in the modification of composition, structure and stability of cell membranes, resulting in membrane dysfunction.[34] Our reports are similar with earlier reports. The high phospholipid level in the kidney of alcohol-fed rats may be due to augmented synthesis or increased free fatty acid levels. The phospholipid level was increased in group II (At) and group IV (D+At) rats due to free radical production and also due to the abnormal fat metabolism. An increased mobilization of lipids from these tissues or decrease in fatty acid uptake and storage capacity may have caused an increase in serum triglycerides and phospholipids in the tissues. Wei, *et al.*[28] reported high phospholipid content in alcohol-treated diabetics. This could be due to an influence of alcohol on phospholipids in diabetic rats.

In the present study, we reported that triglyceride level was increased in kidney tissue of diabetic rats. Hypertriglyceridemia is a common finding in patients with diabetes mellitus and is responsible for vascular complications.[35] Bruan and Severson[36] has reported that deficiency of lipoprotein lipase (LPL) activity may contribute significantly to the elevation of triglycerides in diabetes. In the current investigation, kidney triglyceride level was significantly elevated with alcohol treatment, which received 2 g of alcohol for a period of one month. Available reports showed that chronic alcohol ingestion results in moderate hypertriglyceridemia.[25] Senthilkumar *et al.*[37] in their studies reported the concentration of triglyceride was significantly higher in alcohol treated animals as compared with those of control rats. Diabetic patients who drink alcohol have high triglyceride level.[38] Fukui *et al.*[30] reported that long term use of alcohol can be associated with increased concentration of triglyceride which may also follow U-shaped curve.[39] In alcohol-treated diabetic rats, we observed high levels of triglycerides, this may be due to abnormalities in lipid metabolism and also excess lipolysis in the tissue and also to a number of factors such as increased availability of free fatty acids. Alcohol produced a significant rise in triglyceride levels indicating hyper triglyceride activity of alcohol treatment in diabetes.

In the current study, the content of renal MDA was significantly increased in STZ induced diabetic rats. Hyperglycemia generates reactive oxygen species which in turn cause lipid peroxidation and membrane damage in this study.[40] Previous studies have reported that lipid peroxidation was increased in liver, kidney, and brain of diabetic rats.[41] Lipid peroxide mediated tissue damages have been observed in type I and type II diabetes. Diabetic rats had significantly higher levels of lipid peroxides in plasma urine and renal proximal tubules. Ha and Kim[42] studies showed that the increased oxidative stress in diabetic kidneys may contribute to the increased content of MDA. Chronic alcohol ingestion elevated the level of thiobarbituric acid reactive substances which reflect extensive lipid peroxidation process in the liver, heart and kidney of rats.[43] In the present study lipid peroxidation was significantly elevated with the consumption of alcohol. The depletion of kidney GSH levels along with increased levels of lipid peroxidation on chronic alcohol fed rats reflected the oxidative injury to the kidney. GSH depletion in the tissue has been reported to be a prime factor in the potentiation of lipid peroxidation.[44] Other investigations have also showed the increased cardiac lipid peroxidation after chronic alcohol intake.[25,45] Where as in alcohol-treated diabetic rats, we observed high level of MDA. The increased kidney MDA level of alcohol-treated diabetic rats suggests peroxidative injury and these may be involved in the increased levels of MDA.

Severe tubular degeneration, degeneration of glomeruli, focal necrosis of tubules, cystic dilatation of tubules and fatty infiltration were observed in the kidney tissuej of diabetic rats and, which might be associated with increased diuresis and renal hypertrophy. The above pathological changes were severe in alcohol-treated diabetic rats. Dilatation of Bowman's capsule, hyaline casts were also noted. The presence of hyaline casts represents the production of more reactive oxygen species and more damage. The histological evidence of alcohol-treated diabetic rats suggests that structural alterations at the end of 30 days are due to diabetic and alcohol stress. Thus in addition to high blood glucose, histopathological observations also supports the perception that alcohol at 2 g/kg produced significant increment of lipid metabolic profiles and there by causing tissue damage to renal tissue in diabetic rats.

The results obtained thus suggest that alcohol consumption during diabetic condition elevates the blood glucose levels, total cholesterol, phospholipids, triglycerides and MDA levels. Further studies are needed to know the effect of alcohol on lipid metabolic profiles in diabetic condition. From the above results it is concluded that consumption of alcohol during diabetic condition is unsafe.
